# Role of MicroRNAs in Hepatocellular Carcinoma

**DOI:** 10.5812/hepatmon.18672

**Published:** 2014-08-01

**Authors:** Zixiang Zhu, Xiangle Zhang, Guoqing Wang, Haixue Zheng

**Affiliations:** 1 State Key Laboratory of Veterinary Etiological Biology, National Foot and Mouth Disease Reference Laboratory, Lanzhou Veterinary Research Institute, Chinese Academy of Agricultural Sciences, Lanzhou, China; 2 College of Veterinary Medicine, Gansu Agricultural University, Lanzhou, China

**Keywords:** Hepatocellular Carcinoma, MicroRNAs, Regulation, Therapeutic Targets

## Abstract

**Context:**

MicroRNAs (miRNAs) are small, noncoding RNAs that play an important role in posttranscriptional gene regulation and function as negative gene regulators. They are an abundant class of RNA, each of which can control hundreds of gene targets and regulate diverse biological processes such as hematopoiesis, organogenesis, apoptosis and cell proliferation. Aberrant miRNA expression contributes to tumorigenesis and cancer progression.

**Evidence Acquisition:**

In this study we provided a summarized review of the most important new data available on hepatocellular carcinoma (HCC)-associated miRNAs. The data were collected through searching the related keywords and were categorized and summarized in different sections.

**Results:**

Researchers have reported that miRNAs can repress the expression of important cancer-related genes and might be helpful in the diagnosis and treatment of cancer. During the past two decades, numerous studies have shown that miRNAs play an essential role in inhibiting HCC via several different pathways. Deregulated miRNAs may contribute to carcinogenesis, indicating that miRNAs can act as tumor suppressors and oncogenes.

**Conclusions:**

In this mini review, we highlight current findings and discuss recent work to determine the contribution of miRNA expression to the maintenance and growth of HCC, thereby providing a significant source of hope that miRNAs could serve as therapeutic targets.

## 1. Context

### 1.1. Hepatocellular Carcinoma

Hepatocellular carcinoma (HCC), the most common primary liver cancer, is the fifth most frequent cancer and the third cause of cancer-related mortality worldwide (1). Worldwide incidence of the disease is over 600,000 cases each year (2, 3). HCC normally develops as a consequence of underlying liver disease and is often associated with cirrhosis (4). Incidence is increasing in patients with cirrhosis and other patient subgroups, such as those with human immunodeficiency virus infection or thalassemia (5, 6). Hepatitis B (*HBV*) and hepatitis C (HCV) viral infections, the major risk factors for HCC development, lead to liver cirrhosis and account for 75% of HCC cases (7, 8). Other etiologic factors mainly include toxic chemical exposure, alcohol abuse and intake of aflatoxin-contaminated food (9, 10).

Currently, there are no well-established effective adjuvant therapies for HCC and control of HCC at the initial stage is the most effective therapeutic strategy available. However, this cancer is difficult to diagnose and confirm at the initial stage. In addition, prognosis remains poor for high tumor recurrence and tumor progression. Thus, there is an urgent need to understand the molecular carcinogenic mechanisms of HCC. New research should in addition be focused on developing more therapeutic strategies against HCC. MicroRNAs (miRNAs) have been widely reported to be involved in the development of HCC. Hence, it may be a new target for HCC therapy. In this mini-review, we will discuss some significant miRNAs involved in HCC development with the aim of gaining a better understanding of how miRNAs affect HCC development.

### 1.2. Characteristic of miRNAs

miRNAs are small (21-23 nucleotides), noncoding RNA molecules that down-regulate gene expression by base pairing with 3′ untranslated regions (3′ UTRs) of target messenger RNAs (mRNAs) (11, 12). Lee et al. discovered the lin-4 family in 1993 (13), Since hundreds of similar miRNAs have been identified in plants, animals and viruses through molecular cloning and bioinformatic approaches (14, 15). These findings suggest that miRNAs are a large family of post-transcriptional regulators and control many developmental and cellular processes in eukaryotic organisms (16).

Studies have shown that miRNAs play an important role in the physiological process of animals and plants, such as growth, development, differentiation and reproduction, and that abnormal expression is closely related to human disease (17, 18). miRNAs have emerged as important players in tumorigenesis, which has led to a paradigm shift in oncology. miRNA microarrays have been used to demonstrate that a number of miRNAs function as potential biomarkers for cancer (19). miRNA profiles reflect the developmental lineage and differentiation stages of tumors (11). Many miRNAs have been identified to act as oncogenes, tumor suppressors, or modulators of cancer stem cells and metastasis (20, 21). The rapid discovery of many miRNA targets and their relevant pathways has contributed to the development of miRNA-based therapeutics.

### 1.3. miRNAs and HCC

HCC, a common cause of primary malignant tumors, is a multifactorial disease influenced by a variety of risk factors. Although a number of cellular phenomena (such as inflammation and oxidative stress) and molecular events that facilitate tumor initiation, progression and metastasis have been confirmed (22, 23), the exact mechanism of HCC has not yet been fully elucidated. miRNAs, a class of molecules that regulates post-transcriptional expression, play an important role in tumorigenesis. The discovery of miRNAs and the emergence of corresponding molecular-targeting therapies have added a new dimension to our efforts for combating HCC.

Recent advances in the study of liver miRNAs using gene-modified mice and in vivo nucleic acid delivery for over-expression of specific miRNAs or inhibition of miRNA function have revealed crucial biological roles for individual miRNAs in physiologically essential liver functions in vivo (24). A number of miRNAs have been shown to be aberrantly expressed during liver cancer development (25, 26). In addition, some of these miRNAs might have prognostic significance (27). The aim of this mini review is to discuss current results regarding the molecular aspects of the biological roles of miRNAs in the liver and to explore their relevance to the major and significant molecular mechanisms of cancer occurrence and development.

## 2. Evidence Acquisition

In the present study, we provided a summarized review of the most important new data available on hepatocellular carcinoma (HCC)-associated miRNAs. The data were collected through searching the following related keywords and their different combinations: HCC, miRNA, miRNA regulation, Therapeutic targets, miRNA diagnosis and Carcinoma miRNA. The identified full-text articles were reviewed and the data were summarized in different sections.

## 3. Results

### 3.1. miRNAs Down-Regulated in HCC

#### 3.1.1. miR-122

miR-122, a 22-nucleotide miRNA, is a liver-specific, non-coding polyadenylated RNA that accounts for 70% of the liver miRNA population (28). The miR-122 sequence, transcribed from the *α-helix coiled coil rod homolog (hcr)* gene and the adjacent secondary structure in the *hcr *mRNA are conserved in a wide range of species, including mammals and fish (29). miR-122 is a highly abundant mRNA and is found in human liver, human primary hepatocytes and cultured liver-derived cells, such as the human Huh7 line (30, 31), but is undetectable in other tissues (32). Levels of miR-122 in mouse livers increase to half maximal values around day 17 of embryogenesis and reach near maximal levels (50,000 copies per average cell) prior to birth. Studies demonstrating that expression is specifically induced in mouse liver during embryogenesis suggest that miR-122 is involved in liver development (33). Now that it has been established that the miR-122 was significantly involved in liver development, will *HBV* or *HCV *sequester miR-122 for the HCC development? Studies that address this and other questions should be performed in the future.

Using computational tools, at least two genes have been predicted to be targets of miR-122 and are of interest in tumorigenesis: the N-Myc gene, which is frequently rearranged in woodchuck liver tumors by the woodchuck hepatitis virus (34), the gene referred to as “down-regulated in liver malignancy” (35) and the *cyclin G1* gene (36). The rearrangement or decrease of these genes has been suggested to facilitate HCC development (34, 36).

Numerous studies have reported that miR-122 is down-regulated in human HCCs (36-39). Esau et al. found miR-122 to be a key regulator of cholesterol and fatty-acid metabolism in the adult liver (40). Czech et al. showed that silencing miR-122 resulted in a notable decrease in plasma cholesterol levels, which was consistently associated with a decreased expression of genes involved in cholesterol biosynthesis (41). It revealed the significant role of miR-122 in liver cells. Bai et al. reported that liver-specific miR-122 is frequently suppressed in primary HCC (39). Lin et al. found *B-cell lymphoma 2 like protein 2 (Bcl-w)*, an antiapoptotic gene, to be a direct target of miR-122 that functions as an endogenous apoptosis regulator in HCC-derived cell lines (42). Fornari et al. proved that low miR-122 levels in HCC patients were associated with a shorter recurrence time (43). All of these results suggest that miR-122 is significantly associated with hepatocarcinogenesis and that miR-122 may promote HCC development. Furthermore, a recent study showed that over-expression of miR-122 at an appropriate stage could promote hepatic differentiation and maturation by regulating the balance between proliferation and differentiation through a miR-122–FoxA1–HNF4a-positive feedback loop (44). It therefore appears that miR-122 is a favored miRNA for HCC. The down-regulation of miR-122 may be regulated by host defending systems and the decrease in miR-122 may limit the expansion of HCC. Some studies have also reported controversial changes to miR-122 in HCC, for example, it’s reported a significantly increased level of miR-122 in HCV-associated cancers when compared to non-cancerous tissue (20). We compared these studies and deduced that the different results may have been caused by the diverse detection methods or different samples chosen for analysis. The gender of the patients or the different clinical relevance of HCC may also have resulted in different results. However, to date, most studies have highlighted the decreased expression levels of miR-122 in HCCs compared to normal tissues. All of these results suggest that miR-122 may be an attractive therapeutic target for metabolic disease and as such, provide a significant source of hope that miRNAs could potentially serve as therapeutic targets.

#### 3.1.2. miR-101

miR-101 is a tumor-suppressive miRNA. It is significantly under-expressed in multiple types of cancers, including prostate (45), gastric (46), lung (47, 48), colon (49) and liver cancer (50), and displays a suppressive effect on cellular proliferation, migration and invasion (51). It appears that the host cells assign positive roles to miR-101; however, viruses that can induce tumor occurrence will surely antagonize the expression of miR-101 to enhance its own replication and the decreased expression of miR-101 in tumors may be an achievement on the part of viruses in their bid to defeat host cellular systems.

A study has indicated that miR-101 expression is frequently reduced in human HCC tissues and hepatoma cell lines (50). It is widely known that miRNAs exert their function through regulating the expression of their downstream target genes (12). *Myeloid cell leukemia sequence 1 gene (Mcl-1)*, an antiapoptotic member of the B-cell lymphoma 2 (BCL-2) family (52), is a potential miR-101target. Knockdown of *Mcl-1* can sensitize cancer cells to apoptosis induced by different stimuli, such as serum starvation (53) or chemotherapeutic drugs (54). Wei et al. showed that miR-101 was frequently down-regulated in *HBV*-positive HCC tumor tissues when compared with adjacent noncancerous tissues, suggesting that miR-101 may play a tumor-suppressive role in HCC development (55). Of note, it was found that miR-101 expression inversely correlated with DNA *(cytosine-5-)-methyltransferase 3 alpha (DNMT3A)* expression in HCC and significant down-regulation of miR-101 resulted in DNMT3A up-regulation, which likely occurs during HCC development (55). Zhang et al. confirmed that miR-101 directly targets *sex determining region Y related high-mobility group box 9 (SOX9 )* in HCC, suggesting that miR-101 may suppress HCC tumor progression by down-regulating *SOX9* (56). Furthermore, miR-101 inhibits the expression of the FBJ *murine osteosarcoma viral oncogene homolog (FOS)* oncogene post-transcriptionally by binding to the 3′ UTR of the FOS mRNA, thereby reducing hepatocyte growth factor-induced cell invasion and migration (57). This inhibitive activity of miR-101 possibly directly counteracts the development of HCC. Recently, it was demonstrated that miR-101 represses HCC progression by directly targeting the *enhancer of zeste homolog 2 (drosophila) (EZH2)* oncogene and sensitizes liver cancer cells to chemotherapeutic treatment (58). Shen et al. proved that miR-101 functions as a tumor suppressor by regulating abnormal Nemo-like kinase (NLK) activity in the liver (59). It has also been reported that targeted disruption of NLK inhibits tumor cell growth by simultaneous suppression of cyclin D1 and CDK2 in human hepatocellular carcinoma (60), which implies the anti-tumor activity of miR-101. Xu et al. found that autophagy was suppressed by miR-101 in the HepG2 HCC cell line via targets, including *ras-related protein rab-5A (RAB5A)*, *stathmin 1 (STMN1)* and *autophagy related 4D, cysteine peptidase (ATG4D)* (61). Autophagy has been widely reported to facilitate virus replication; the suppression of miR-101 to the appearance of autophagy may indirectly inhibit virus replication.

Overall, the presence of miR-101 may serve as a biochemical marker for monitoring the progression of tumor development in *HBV*-related HCC; moreover, miR-101 may be a potential prognostic marker and therapeutic target for HCC.

#### 3.1.3. miR-124

miR-124 is a brain-enriched miRNA that plays a crucial role in gastrulation and neural development (62, 63). miR-124 has been widely reported to regulate a plethora of target proteins involved in cell cycle, differentiation, cellular development and migration (64, 65). Recent profile studies of miRNA expression have documented a deregulation of miR-124 in HCC (66).

In HCC cell lines, stable over-expression of miR-124 was sufficient for inhibiting cell motility and invasion in vitro, and additionally, suppressed intrahepatic and pulmonary metastasis in vivo (67). This implies that miR-124 can possibly limit the metastasis of HCC. Zheng et al. demonstrated that the expression levels of miR-124 were frequently reduced in HCC cells and tissues, and low-level expression of miR-124 was significantly associated with a more aggressive and/or poor prognostic phenotype of patients with HCC (67). Furuta et al. showed that miR-124 and miR-203 are novel tumor-suppressive miRNAs for HCC that are epigenetically silenced and that activate multiple targets during hepatocarcinogenesis (68). Lang et al. determined that miR-124 functions as a growth-suppressive miRNA and plays an important role in inhibiting tumorigenesis by targeting *phosphatidylinositol-4,5-bisphosphate 3-kinase and catalytic subunit alpha (PIK3CA)* (69). Zeng et al. indicated that low miR-124 levels mediated by *HCV *via *DNA (cytosine-5-)-methyltransferase 1 (DNMT1)* promote interstitial cells of Cajal cell migration and invasion by targeting *SET *and *MYND domain-containing 3 (SMYD3)* (64). Lu et al. showed that miR-124 functions as a tumor suppressor in HCC by targeting the *signal transducer and activator of transcription 3 (STAT3)* (70). These studies suggest that miR-124 can target various genes in order to suppress HCC development. Conversely, HCC-related viruses, such as *HBV* and *HCV *will antagonize the expression of miR-124 and its activity; decreased expression levels of miR-124 may be the result of counteracting by the viruses, however, more studies should be developed in order to better understand this counteracting process.

In summary, the data discussed above highlight an important role for miR-124 in the regulation of invasion and metastasis in the molecular etiology of HCC and suggest the potential application of miR-124 in prognosis prediction and cancer treatment.

#### 3.1.4. Let-7 Family

The let-7 miRNA family has 13 members located on nine different human chromosomes. The family was first discovered in *Caenorhabditis elegans *and is functionally conserved in worms and humans (71). It is widely viewed as a tumor suppressor miRNA. Many human cancers exhibit deregulated let-7 expression when compared to normal tissue (72-74).

Several studies have suggested that *lin28s *may promote transformation by targeting let-7 and that the intricate balance between *lin28* and let-7 may be critical for regulating tumor development and progression (75, 76). Johnson et al. demonstrated that human let-7 specifically targets *rat sarcoma (RAS)* in human cancer cells (77), which was confirmed in non-small cell lung cancer, using a mouse model in which let-7g inhibited tumor growth via the suppression of *RAS* (78). Recently, several studies have identified the role of let-7 in HCC development. Using microarray analysis, Shimizu et al. found that let-7 miRNAs negatively regulate *B-cell lymphoma 2 like protein 1 (Bcl-Xl)* expression and can induce apoptosis when used with an anti-cancer drug targeting *myeloid cell leukemia sequence 1* (*Mcl-1*) in human HCC (79). It’s identified that let-7g may partially suppress HCC metastasis through targeting *collagen, type I, alpha 2 (COL1A2)* (80). The various functions of the let-7 miRNA family involved in DNA replication, apoptosis and cellular differentiation create a collaborative inhibitive effect against HCC development. Hence, restoration of let-7 expression may be a useful therapeutic option for HCC where let-7 expression is absent.

### 3.2. miRNAs Up-Regulated in HCC

#### 3.2.1. miR-221

miR-221 has been reported to be over-expressed in many human tumor tissues, such as breast cancer (81), colorectal cancer (80), glioblastoma (82) and prostate cancer (83). A recent report shows that miR-221 stimulates the onset of tumors and promotes tumor progression, significantly shortening the mean time to death in a mouse model of liver cancer (84). The promotive roles of miR-221 in tumor progression may result from the interfering function of miR-221 against some cellular anti-oncogenes. Tumor progression could potentially stimulate the expression of miR-221 and promote its development.

In highly aggressive HCCs, miR-221 was among the most up-regulated of all miRNAs studied. The key miR-221-targeted tumor suppressors include p27, p57, *phosphatase and tensin homolog (PTEN)*, a *tissue inhibitor of metalloproteinase-3* and the *DNA damage-inducible transcript 4 (DDIT4 )* (85-87). Li et al. found miR-221 expression to be correlated with tumor size, cirrhosis and tumor stage. Kaplan-Meier survival analysis showed that the overall survival rate of the high miR-221-expression group was significantly lower than that of the low miR-221-expression group (88), implying that serum miR-221 may provide predictive significance for the prognosis of HCC patients. miR-221 is capable of stimulating tumor growth in vivo, possibly through down-regulation of p27 and/or *DDIT4 *expression. A recent study offers preclinical proof for the efficacy of chol-anti-miR-221 in a valid orthotopic mouse model of HCC, suggesting that this targeted agent could improve treatment for patients with advanced HCC (89). Pineau et al. revealed an important contribution for miR-221 in hepatocarcinogenesis and suggested a role for *DDIT4 *dysregulation in this process (84). The dysregulation roles of miR-221 in cellular apoptosis and differentiation indicate the negative regulatory effect of miR-221. Thus, the use of synthetic miR-221 inhibitors may be a promising approach for HCC treatment.

#### 3.2.2. miR-224

miR-224 is a potential oncogenic miRNA that impacts multiple crucial cellular processes (90). Transfection of miR-124 induced neuronal phenotypic changes by affecting the expression of neuronal-specific markers in stem cells derived from both mouse neural/brain tumor and human glioblastoma multiforme (90). miR-124 is greatly up-regulated in human HCC when compared to both paired peritumoral cirrhotic tissues and cirrhotic livers without HCC (91), and plays a role in cell proliferation, migration, invasion and anti-apoptosis (92). It has also been reported that the expression of miR-224 and the host *gamma-aminobutyric acid (GABA)* *A receptor, epsilon (GABRE)* gene (located on human chromosome band Xq28) increased progressively as normal liver progressed to a benign hepatocellular state (93). Taken together, these results imply a co-carcinogenic role for miR-124.

Histone acetylation was found to play a partial role in the regulation of miR-224 and genes at the Xq28 locus in patients with HCC (94). Wang et al. have shown that miR-224 is coordinately up-regulated with neighboring miR-452 and genes at Xq28 in HCC tumors (94). Scisciani et al. identified that p65/NF-κB (nuclear factor kappa-light-chain-enhancer of activated B cells) is a direct transcriptional regulator of miR-224 expression and links miR-224 up-regulation with the activation of the LPS, LTα, and TNFα inflammatory pathways, as well as cell migration/invasion in HCC (95). The negative regulation of miR-224 on NF-κB or other cytokines expression may directly block the anti-tumoral ability of the host, causing the involved pathways to shift to abnormal status and finally facilitate HCC development. Taken together, these studies suggest that miR-224 may represent a promising biomarker for assessing liver status in the case of liver disease and HCC. Some synthetic medicines against miR-124 may potentially suppress or alleviate the development of HCC.

#### 3.2.3. miR-21

miR-21 aberrant expression was first identified by miRNA profiling of human glioblastoma (96). Subsequently, miR-21 has become one of the most studied miRNAs associated with cancer. miR-21 has been reported to be up-regulated in various types of tumors and it plays an important role in cancer pathogenesis and progression (97). Asangani et al. reported that miR-21 induces invasion, intravasation and metastasis (98). It was significantly up-regulated in HCC tissues and cell lines, compared with adjacent non-tumor tissues and normal hepatic cells. miR-21 up-regulation is associated with the capacity of tumor migration and invasion in HCC. The targets of miR-21 may involve some tumor suppressive factors; therefore, the up-regulation of miR-21 can potentially result in the decrease of some anti-tumor proteins, which finally promote the development of HCC. The relationship between various anti-tumor factors and miR-21 should be studied in future to better understand the roles of miR-21 in HCC development.

Since the aberrant expression of miR-21 was confirmed in the miRNA profiling of human glioblastoma (96), miR-21 has been identified as one of the most important miRNAs dysregulated in cancer, including breast (99), colorectal (98), gastric (97), lung (100) and liver (101) cancers. Meng et al. showed that miR-21 was highly over-expressed in HCC tumors and cell lines in expression-profiling studies using miRNA microarrays. Inhibition of miR-21 in cultured HCC cells increased expression of the *phosphatase and* *tensin homolog (PTEN)* tumor suppressor, and decreased tumor cell proliferation, migration and invasion (102). Whether *PTEN* is one of miR-21 targeted genes, or if one of the upstream regulatory genes of *PTEN* is a target gene of miR-21, these genes should be further studied in future.

In both human and woodchuck HCC cell lines, separate treatments with antisense oligonucleotides specific to miR-21 caused a 50% reduction in both hepatocyte proliferation and anchorage-independent growth (103). These observations suggest that new drugs or antisense oligonucleotides specific to miR-21 may be considered a potential therapeutic strategy for HCC.

## 4. Conclusions

HCC is one of the most common primary liver cancers and is a major global health problem that sees an increasing number of new cases diagnosed each year. miRNAs play an important role in physiological processes, many of which are involved in hepatocarcinogenesis. Many studies have reported the identification of miRNA biomarkers, their target genes and the possible mechanisms that lead to hepatocarcinogenesis. miR-124 can be chosen as a prognostic factor for HCC occurrence and the upregulation of its target gene *DNMT1* may also be applied as a prognosis for HCC development. Some inhibitory medicines against miRNAs that can facilitate HCC development can possibly also be used in HCC therapy in future. The roles of some particular miRNAs are listed in Table 1, which provide references for further study concerning HCC and miRNAs. Understanding the role of miRNAs in the biology of HCC can potentially provide advances and options for HCC treatment, and might be useful for HCC diagnosis. Future research is needed to address and extend the therapeutic potential of miRNAs in inhibiting the progression of HCC. Additionally, miRNAs can provide new sight and targets for the development and research of new medicines against HCC.

**Table 1.** miRNAs Associated With HCC Development and Their Target Genes

Expression levels’ change in HCC	miRNAs	Association with HCC development	Target genes
**Down-regulation**	miR-122	Controversial	*N-Myc; Cyclin G1; Bcl-w; ADAM17; Wnt1*
	miR-101	Suppressive	*Mcl-1; EZH2; EED;DNMT3A; SOX9*
	miR-124	Suppressive	*PIK3CA; STAT3; SNAI2*
	let-7 family	Suppressive	*Caspase-3; HMGA2; Bcl-xL; c-myc*
	miR-125a	Suppressive	*MMP11; VEGF-A; SIRT7*
	miR-125b	Suppressive	*LIN28B2; PIGF; Bcl-2*
	miR-203	Suppressive	*c-JUN; SOCS3*
	miR-139	Suppressive	*ROCK2; c-Fos*
	miR-1	Suppressive	*ET-1; HSP60*
	miR-145	Suppressive	*IRS1; IRS2; Oct4*
	miR-138	Suppressive	*CCND3; FAK*
	miR-7	Suppressive	*PIK3CD; mTOR; p70S6K*
**Up-regulation**	miR-221	Promotive	*CDKN1C/p57; DDIT4;Arnt*
	miR-224	Promotive	*RKIP; CDC42; CDH1;PAK2; BCL-2; MAPK1;API-5*
	miR-21	Promotive	*PTEN; RHOB; PDCD4*
	miR-155	Promotive	*APC; AT1R; SHIP1*
	miR-143	Suppressive	*FNDC3B; Bcl-2*
	miR-373	Promotive	*PPP6C; TXNIP; RABEP1*
	miR-210	Unclear	*VMP1;AIFM3*
	miR-182	Promotive	*MTSS1; CYLD; Foxo1*
	miR-181b	Suppressive	*TIMP3; Mcl-1*
	miR-519d	Promotive	*CDKN1A/p21; PTEN; AKT3; TIMP2*
	miR-550a	Promotive	*CPEB4; AKT1*
	miR-657	Promotive	*TLE1; IGF2R*
